# Prioritizing Mentorship as Scientific Leaders

**DOI:** 10.1021/acscentsci.3c00500

**Published:** 2024-01-10

**Authors:** Jacky M. Deng, Salma Elgaili Ahmed, Ernest Awoonor-Williams, Progna Banerjee, Magda H. Barecka, Laura E. Bickerton, Silvina A. Di Pietro, Stanna K. Dorn, Kevin Maik Jablonka, Gabriele Laudadio, Elisabeth Kreidt, Helena Mannochio-Russo, Júlio Terra, Olivia Harper Wilkins, Saigopalakrishna S. Yerneni, Maha Yusuf

Sustaining innovation in science, technology, engineering, mathematics, and medicine (STEMM) requires collaborative problem-solving, particularly as we work to address complex global issues.^1^ Effective mentoring strategies are essential in accomplishing this goal by helping support diverse talent that will strengthen the future of STEMM.^2^ Multiple studies have shown that mentorship is positively related to graduate students’ persistence in research productivity, research self-efficacy, rate of degree completion, and program satisfaction.^3−6^

Despite its importance, mentorship is often an overlooked and underappreciated component of scientific training. In this article, we—a diverse authorship team representing ten countries and various chemistry subdisciplines brought together by the CAS Future Leaders program—share mentorship experiences and actions that readers can use to promote more inclusive and productive mentor-mentee relationships in chemistry. We share our insights by reflecting on three key questions:

(1) Why is mentorship important?
(2) What have been some impactful mentorship strategies we have used or experienced?
(3) How can we create a chemistry community that values and prioritizes effective mentorship?

The following sections share patterns across our diverse experiences and perspectives. Full responses from the authors can be found in the Supporting Information.

## 1 Why Is Mentorship Important?

**Figure 1:**
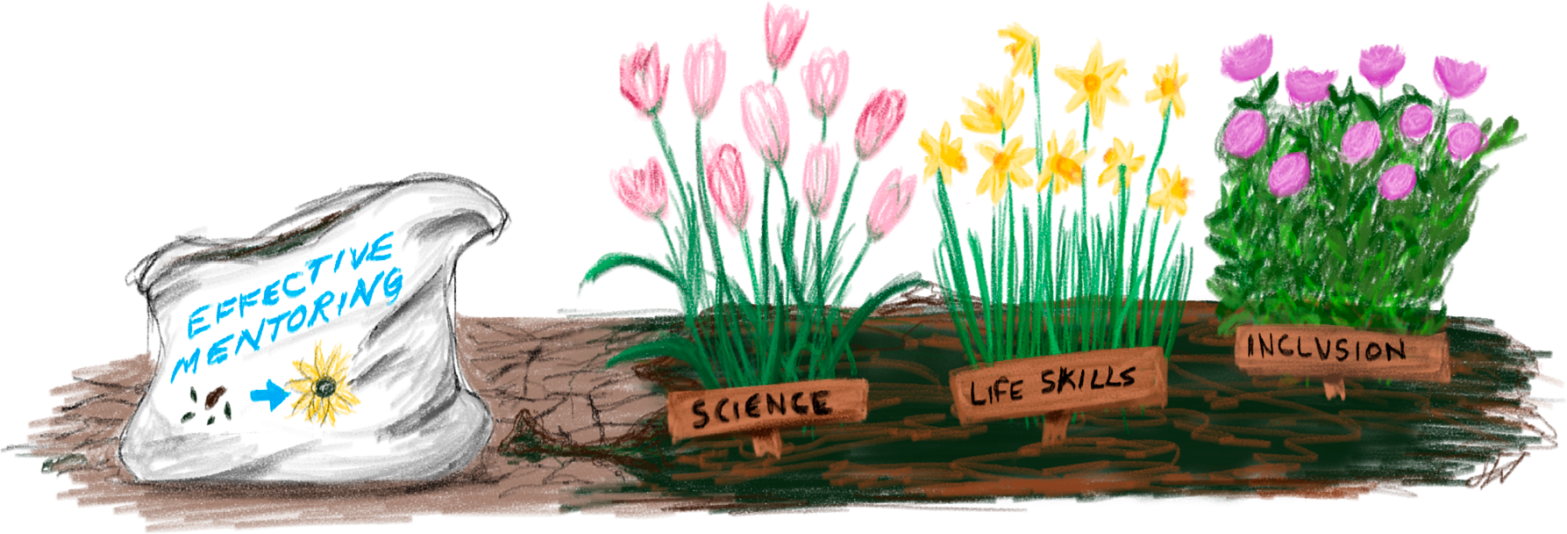
Effective mentorship fosters not only growth in science but professional and personal skills and inclusion. Figure created by Olivia Harper Wilkins.

Reflecting on our collective experiences, our responses to this question identified the following themes. (1) Mentors play a vital role in equipping mentees with key *scientific* skills, such as experimental techniques or how to think critically about their research and careers. (2) Mentors are also crucial in helping mentees develop *professional* skills, such as networking and navigating careers. (3) Lastly, mentors play a critical role in supporting mentees in navigating research challenges and developing resilience (Figure 1).

### 1.1 Mentors Teach Us How to Be Scientists

#### 1.1.1 Building Our Scientific Skills

Scientific mentors play a vital role in imparting essential tacit knowledge concerning research methodologies, experimental design, and data analysis. Through their guidance, mentees can cultivate a solid foundation for conducting research.

#### 1.1.2 Fostering Our Scientific Thinking

Mentors not only facilitate the acquisition of scientific skills but also cultivate a self-belief in mentees that they are scientists. They inspire mentees to embrace critical thinking, pose questions, and challenge assumptions. This process equips mentees with the capacity to assess existing knowledge, pinpoint gaps, and propose innovative experimental approaches. Furthermore, effective mentors can contribute to the development of problem-solving proficiency by sharing their personal experiences in overcoming research hurdles.

### 1.2 Mentors Teach Us More than Science

#### 1.2.1 Supporting Our Personal and Professional Development

Scientific mentors extend their guidance beyond scientific knowledge, offering valuable insights into “soft” skills, such as effective communication, presentation techniques, and scientific writing. They can impart valuable life skills, such as time management, goal setting, or modeling a growth mindset when challenges arise.

#### 1.2.2 Helping Us Navigate Career Paths

Mentors play a pivotal role in helping mentees navigate the range of career choices, both within academia and beyond. Their experience can significantly help with decisions related to pursuing advanced degrees, transitioning into industry roles, or engaging in science policy.^7^ Mentors can also help mentees discover diverse career opportunities by facilitating introductions to their professional network, collaboration opportunities, and exposure to different research environments and perspectives.

### 1.3 Mentors Can Influence Who Continues in Science

#### 1.3.1 Inspiring and Motivating Mentees

Science and research rarely follow a straightforward, linear path. Mentors serve as role models for their mentees, and their approach and attitude toward science, as well as their ability to support and encourage mentees, can motivate individuals to pursue and persist in science.

#### 1.3.2 Supporting Scientists from Historically Underserved Groups

Mentors play a vital role in supporting individuals from historically underserved groups in science. They can offer the essential support, understanding, and guidance that are needed to overcome barriers and biases in the scientific community (e.g., the hidden curriculum, implicit expectations). Their role extends to advocating for systemic changes that work to disrupt and dismantle inequities faced by underserved groups, contributing to a more inclusive scientific community.

## 2 What Have Been Some Impactful Mentorship Strategies We Have Used or Experienced?

Every mentoring relationship is unique. However, from our experiences, certain strategies can serve as a foundation for fostering effective mentor-mentee interactions. These include (1) setting clear expectations, (2) establishing rapport, (3) maintaining open communication, and (4) empowering mentees.^3^

### 2.1 Aligning Expectations

#### 2.1.1 Defining Roles and Responsibilities

We all found that it was important to have a conversation at the start of our mentorship relationships to outline what each person’s roles and responsibilities were. Our most effective mentors have always clarified early on how often we would meet, what kind of feedback they would provide, and what we, as mentees, were expected to contribute.

#### 2.1.2 Setting Clear Timelines and Goals

Establish specific, measurable, achievable, relevant, and time-bound (SMART) goals, such as setting specific timelines for completing a literature review or reaching a specific phase of a project. For example, when handling lab projects, we worked with our mentors to set a goal for completing the initial experiments within an agreed-upon time period. Having these time-bound goals keeps everyone on track.

#### 2.1.3 Discussing Short- and Long-Term Goals

Plan sessions to talk about both immediate and future goals. For example, one could discuss the short-term goal of completing a literature review by the end of the month and the long-term goal of presenting findings at a conference in six months. This way, everyone is aware of and working toward the same objectives.

#### 2.1.4 Track Progress Together

Keep a shared document or tool to track progress collaboratively. This could be a simple spreadsheet or document where you note what tasks have been completed, what’s in progress, and what’s coming up. This real-time tracking has helped us and our mentors stay informed about our progress.

### 2.2 Building Rapport

#### 2.2.1 Choosing Kindness

Regardless of any other label we may give ourselves, we are human beings first. Our most effective mentors have always prioritized empathy and kindness. If you’re comfortable, invite opportunities for conversations about personal experiences and interests (e.g., hobbies, interests, values). This personal touch cultivates trust and enriches the mentor-mentee relationship. Attending professional (e.g., seminars, workshops) and group events together can also help strengthen such bonds.

#### 2.2.2 Sharing Failures

In research, we place so much emphasis on people being “experts” in something that sometimes we forget that we can’t be experts in all areas. Our best mentors have reminded us that we are not expected to know everything (or perhaps even anything). This has helped us feel more secure in our pursuit of knowledge, empowering us to spend our energy stumbling through research rather than pretending we are going through the process flawlessly. It helps when mentors open up about their own past challenges and failures in their research journey, for example, discussing a project that didn’t go as planned or an experiment that didn’t yield the expected results. This helps mentees understand that setbacks are normal and encourages them to approach research with a growth mindset.

#### 2.2.3 Encouraging Questions

Creating an environment where asking questions is encouraged and that curiosity drives scientific progress. As mentees, some of us may be hesitant to ask a question. Effective mentors recognize this and actively invite mentees to share what they’re thinking. This has helped us move past viewing questions as a sign of weakness but rather as an opportunity for growth.

### 2.3 Maintaining Open Communication

#### 2.3.1 Scheduling Regular Check-ins

A brief, regular, agreed-upon check-in period to discuss progress and address any challenges can help maintain open communication between the mentor and the mentee. Consistent communication also helps mentees feel supported.

#### 2.3.2 Listening Actively

Practice active listening by showing genuine interest in mentees’ thoughts and concerns. This demonstrates empathy and contributes to building a strong rapport.

#### 2.3.3 Promoting a Culture of Constructive Feedback

Mentorship is a two-way street. Our mentees have given us opportunities to provide feedback on the mentorship experience, helping us establish a two-way communication channel and culture of growth. For instance, they have asked us for our perspective on what aspects of the experience have been helpful and what could be improved. Surveys could also be used to mitigate mentor–mentee power dynamics, allowing mentees to provide their feedback anonymously.

### 2.4 Facilitating Mentee Agency

#### 2.4.1 Supporting Self-Reflection

Our mentors have assigned us self-reflection exercises where, rather than having them evaluate us, we evaluate our own progress, strengths, and goals. These can take the form of regular progress reports/updates in which mentors prompt their mentees to reflect on their professional and personal development after a certain period of time, followed by a 1-on-1 discussion.

#### 2.4.2 Encouraging Autonomy and Independence

Our most effective mentors recognized when we were ready for the opportunity to lead projects or experiments. Allowing us to make decisions and take ownership fostered a sense of agency and boosted our confidence. For example, mentors can give mentees independent opportunities to explore the literature, tools, and potential collaborators. If one is mentoring a student on a lab project, invite them to take the lead on planning and executing certain experiments.

## 3 How Can We Create a Chemistry Community That Values and Prioritizes Effective Mentorship?

Although mentorship is crucial for retaining talent and promoting progress in chemistry, it often receives less attention compared to other areas of our profession and culture.^8^ Cultural change and community buy-in are needed, which can be accomplished through a systemic approach to incorporating and incentivizing effective mentorship in all aspects of our scientific community and practice.^9,10^ We emphasize the importance of highlighting and valuing mentorship through mentorship awards and including mentorship as criterion in applications for scientific scholarships, awards, grants, and tenure applications. There must also be opportunities for scientists to regularly discuss, practice, and reflect on mentorship alongside colleagues and peers. Lastly, broader policy changes are needed to ensure that how our community views and values mentorship is sustained and formalized.

### 3.1 Highlighting Effective Mentorship

#### 3.1.1 Recognizing Outstanding Mentors

Establishing awards or recognition programs that celebrate mentors who have made significant positive impacts on their mentees’ careers and growth. For instance, a departmental award could include a category for exceptional mentorship. Additionally, consider integrating mentorship evaluation into existing milestones such as research awards or tenure assessments. Regularly feature mentor profiles, success stories, and interviews in community newsletters, journals, or social media platforms. These mechanisms and initiatives emphasize the importance of mentorship within our chemistry community.

#### 3.1.2 Evaluating Mentorship As a Criterion in Award, Grant, and Job Applications

Explicitly including mentorship as a criterion in award, grant, job, and other applications communicates to members of our chemistry community that mentorship is something we prioritize and view as an *essential* part of being successful scientists. This may also help our community better recognize the contributions members of historically underrepresented groups in science, who have historically and disproportionately been called upon to mentor and support students and trainees.^11^ Ways to do this include asking for mentorship statements (perhaps included as part of teaching statements) or inviting reference letters from mentees.

#### 3.1.3 Hosting Panel Discussions and Webinars

Organizing panel discussions and webinars that focus on successful mentorship stories and strategies. These events can showcase the impact of mentorship and provide insights into effective practices. For instance, a panel could discuss how mentorship aided a mentee’s transition from academia to industry and the strategies that contributed to that success.

### 3.2 Creating Opportunities to Learn and Practice Mentorship

#### 3.2.1 Creating Mentorship Training Workshops

Host hands-on workshops that provide mentors with practical training on effective mentorship techniques, such as the skills described in this article. These could also include role-playing scenarios where mentors and mentees can practice setting expectations/goals, having challenging conversations, or providing feedback. One example of this is the ACS Mentor Training Workshop for Graduate Students and Postdoctoral Scholars, which helps early career researchers “learn strategies for developing or improving mentoring skills to cultivate productive mentor-mentee relationships”.^12^ Other examples include the ACS’s Postdoc to Faculty and New Faculty Workshops and the RSC’s Career Talks.^13−15^ Additional resources and workshops are available in the Supporting Information (Laura; pp 20–21).

#### 3.2.2 Establishing Peer Mentorship Circles

Establish small, supportive peer mentorship circles. These circles serve as spaces where mentors and potential mentors come together to discuss hurdles, share personal experiences, and collectively work on their mentorship skills. Through candid conversations, mentors can learn from each other’s insights and adapt their strategies accordingly. For instance, a circle could meet monthly to discuss common challenges, compare strategies, and brainstorm effective solutions.

#### 3.2.3 Fostering Diverse Mentor-Mentee Matchmaking

Mentor-mentee matching programs within the chemistry community can help create structured opportunities for mentors and mentees to connect. One example of this is the ACS Career Consultants program, which allows individuals to connect 1-on-1 with experienced ACS members who are willing to share their expertise in specific job markets (academia/industry), processes (such as transitioning into an independent research position or successful retirement), and their passion in seeing the long-term growth and success of their mentees.^16^ Other examples include departmental mentorship programs and the Chemistry Women Mentoring Network (CWMN).^17,18^

### 3.3 Advocating for Policy Changes That Prioritize Mentorship

In order for effective mentorship to be sustained and practiced, it must also be formalized through policy changes. Many of the strategies discussed in this article are aligned with developing individual development plans (IDPs): a systematic approach for helping individuals in their professional growth and career advancement. Required, annual IDPs are becoming increasingly common in graduate programs,^19,20^ and their use is reviewed in grant applications for the NIH.^21^ These types of policy changes that ensure mentors and mentees have regular conversations around expectations, progress, goals, and career development could help our community begin to view mentorship as an essential part of our profession. ACS and AAAS/*Science* have also developed online tools to support graduate students and postdoctoral researchers in developing IDPs.^22,23^

#### 3.3.1 A Call to Action: Prioritizing Mentorship As Scientific Leaders

Mentorship is an essential aspect of success in science, yet it is often overlooked. To establish a successful mentorship relationship, both mentors and mentees must set clear goals and expectations, build rapport, and facilitate open communication.

How can we make mentorship a more normalized topic in academia and beyond? By engaging in discussions about the advantages of mentorship, we can emphasize its importance as a skill to be developed. And it’s not just mentees who benefit—mentors can also gain valuable insights and experiences through the process.

To encourage and facilitate mentorship, organizations should establish programs and awards that incentivize and recognize effective mentoring. By making mentorship a priority and investing in functional mentoring alliances, we can create a culture of growth and development that benefits everyone involved.

**Figure 2:**
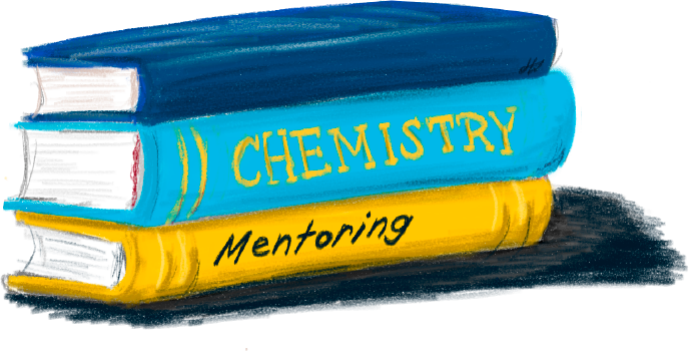
We believe mentoring is an essential component of STEMM education, and we hope that mentorship programs will become a regular part of professional training. Figure created by Olivia Harper Wilkins.

As early career researchers in the chemical sciences, we believe that effective mentorship is foundational for sustained innovation in STEMM fields (Figure 2). We hope to inspire readers to prioritize mentorship in their scientific careers and to recognize its importance through tangible actions. Together, we can create a culture of inclusivity and support that strengthens the future of chemistry.

## Supporting Information Available

Full responses to mentorship questions from authors and author biographies (PDF)

## ABBREVIATIONS

STEMM: science, technology, engineering, mathematics, medicine
RSC: Royal Society of Chemistry
AAAS: American Association for the Advancement of Science
NIH: National Institutes of Health
