# Supplementary material for: Prioritizing Mentorship as Scientific Leaders
Source: ACS Cent Sci. 2024 Jan 10;10(2):209–13. doi: 10.1021/acscentsci.3c00500 (PMC10906028; doi:10.1021/acscentsci.3c00500)
Supplement: Supplementary file 1 — oc3c00500_si_001.pdf [file oc3c00500_si_001.pdf]

Supporting Information: Prioritizing Mentorship as Scientific Leaders

Jacky M. Deng[a,*], Salma Elgaili Ahmed[b,‡], Ernest Awoonor-Williams[c,‡], Progna Banerjee[d,‡], Magda Barecka[e,‡], Laura E. Bickerton[f,‡], Silvina A. Di Pietro[g,‡], Stanna K. Dorn[h,‡], Kevin Maik Jablonka[i,j‡], Gabriele Laudadio[k,‡], Elisabeth Kreidt[l,‡], Helena Mannochio-Russo[m,‡], Júlio Terra[n,‡], Olivia Harper Wilkins[o,‡], Saigopalakrishna S. Yerneni[p,‡], Maha Yusuf[q,‡]

AUTHOR ADDRESS: (a) Department of Chemistry and Biomolecular Sciences, University of Ottawa, Ottawa, ON K1N 6N5, Canada; (b) Centre for Advanced Imaging and The Australian Institute for Bioengineering and Nanotechnology, The University of Queensland, St Lucia, Qld 4072, Australia; (c) Novartis Institutes for BioMedical Research, 181 Massachusetts Avenue, Cambridge, Massachusetts 02139, United States; (d) Center for Nanoscale Materials, Argonne National Laboratory, Argonne, Illinois 60439, USA; (e) 1. Department of Chemical Engineering, 360 Huntington Avenue, 02215 Boston, MA, 2. Department of Chemistry and Chemical Biology, 360 Huntington Avenue, 02215 Boston, MA; (f) EaStCHEM School of Chemistry, University of Edinburgh, Joseph Black Building, David Brewster Road, Edinburgh, Scotland EH9 3FJ, United Kingdom; (g) Physical and Life Sciences Directorate, Lawrence Livermore National Laboratory, Livermore, California 94550, United States; (h) Division of Chemistry and Chemical Engineering, California Institute of Technology, Pasadena, California 91125, United States; (i) Laboratory of Organic and Macromolecular Chemistry (IOMC), Friedrich Schiller University Jena, Humboldtstrasse 10, 07743 Jena, Germany; (j) Center for Energy and Environmental Chemistry Jena (CEEC Jena), Friedrich Schiller University Jena, Philosophenweg 7a, 07743 Jena, Germany (k) Department of Chemistry, Scripps Research, La Jolla, California 92037, United States; (l) Department of Chemistry and Chemical Biology, TU Dortmund University, Otto-Hahn-Straße 6, Dortmund 44227, Germany; (m) Skaggs School of Pharmacy and Pharmaceutical Sciences, University of California San Diego, La Jolla, CA 92093, USA; (n) Laboratory of Sustainable and Catalytic Processing, Institute of Chemical Sciences and Engineering, École Polytechnique Fédérale de Lausanne (EPFL), Station 6, Lausanne, 1015 Switzerland; (o) NASA Postdoctoral Program Fellow, NASA Goddard Space Flight Center, 8800 Greenbelt Road, Greenbelt MD 20771; (p) Department of Chemical Engineering, Carnegie Mellon University, Pittsburgh, Pennsylvania 15213, USA; (q) Department of Chemical Engineering, Stanford University, CA 94305, USA

Author Contributions
*Corresponding author
‡Authors contributed equally to this work.

1. Why is mentorship important?

Progna: As an early career researcher mentorship is incredibly important to me for several reasons. Mentorship is a key component of my role as an academic and is critical to creating a supportive, collaborative, and productive academic environment.

I believe that it is crucial to give back to the next generation of students and scholars by providing guidance, support, and mentorship. As an academic, I have had the privilege of working with many inspiring mentors who have helped me to develop my research skills, build my professional network, and navigate the complex academic landscape. Without their support and guidance, I would not be where I am today, and I am committed to paying it forward by serving as a mentor to students and junior colleagues. In addition to the satisfaction that comes from helping others, I believe that mentorship is also an important part of building a strong academic community. By fostering relationships with students and junior colleagues, I hope to create a network of scholars who can support each other, collaborate on research projects, and share resources and ideas. This type of collaborative community is essential for building a thriving research program and for making meaningful contributions to the academic field.

Elisabeth: Science is hard. By its very nature it is confronting you with unsolved problems and difficult tasks every day. You need a lot of (technical) knowledge, creativity and stamina to progress, and in many cases, you will lack a bit of one of these resources. This is where you will need somebody who has already progressed a bit more in their science journey, who has already solved a puzzle similar to yours or who can see the bigger picture more clearly. This is where you will need a mentor. As I have experienced it, a good mentor can make a huge contribution to how you face your challenges. Good mentorship often makes the difference between just getting it done "somehow" and extracting valuable personal growth while getting it done. While you are busy working on your everyday challenges, a mentor can help you to keep focused on the bigger picture, stay creative and keep everything in balance with your personality and personal needs. What I've learned from my mentors has become an integral part of my research personality and of how I approach science and I try to give something similar to younger chemists I am working with. Now that I am a PI of a small group, I learn that being a mentor is a complex task that needs a lot of thought and empathy. It is a challenging thing on its own (where you might need a bit of help from your own mentors) but also enormously rewarding as it allows your knowledge and abilities to contribute to more than your own work. You can make a real, sustainable impact. Apart from that the interaction with your mentees brings you fresh ideas and approaches, helping you to not become too stuck to your own established strategies and thoughts.

Ernest: Mentorship is important to me because it provides an opportunity for personal and professional growth, self reflection, and connection. Effective mentorship breeds confidence in mentees — offering guidance, support, and training as mentees navigate different aspects of their lives. Personally, I find mentorship has shaped and challenged my beliefs as a researcher and played a significant role in my scientific identity. Throughout my academic career, I have been fortunate to have great mentors who have not only provided guidance and advice but also challenged me to take ownership of difficult tasks and projects. I believe mentorship is a two-way street; it benefits the mentee as much as it benefits the mentor.

Helena: "Good" mentorship can help flourish the best of someone, make this person feel accomplished, and guide him/her toward a fruitful career. On the other hand, "bad" mentorship can be highly frustrating and negatively impact someone's vision of a specific field, for instance, even leading someone to make a dramatic change in their career. These two simple scenarios exemplify the dramatic impact that mentorship may have on someone's professional and personal life. Mentors are fundamental to guidance and encouragement throughout our scientific journey, and building a healthy and trustworthy mentor-mentee relationship is critical. The most obvious image of a mentor is the formal supervisor or advisor. However, getting advice from people of different levels who have undergone specific experiences of interest is valuable to diversifying the precious inputs someone can have. Having a comprehensive network with people from diverse fields and backgrounds is the first step toward getting a unique way of thinking.

Gabriele: Mentorship is essential in many aspects of science. It is one of the most effective ways to transfer knowledge from the mentor to the mentee, encouraging the development of both soft and hard skills. Through constant communication, the mentor and mentee create a feedback loop that connects them and allows them to share similar experiences. This connection triggers personal and professional growth for both parties.

Mentorship is important to me because it is the main way for scientists to learn and teach in a working setting. I have witnessed how the mentorship process can dramatically enhance productivity and self-awareness, leading to constant evolution that brings both the mentor and the mentee to a more mature stage. Moreover, a fruitful mentor-mentee relationship generates a healthy environment where everyone feels free to discuss, make mistakes, and provide genuine feedback in a respectful manner. In my experience, I have seen how such a relationship boosts the confidence of both parties and makes the work light, fun, and easy. I truly believe that mentorship is the only way to build a solid foundation in our scientific society, where each individual can express their personality freely.

Kevin: Mentorship is important to me because there are many things that cannot be learned from textbooks or conventional means. For example, when transitioning into a new leadership role in a lab, there are many things that are not typically taught, and having a mentor to talk through these issues can be extremely valuable. Additionally, mentors can help with exploring ideas and making career decisions. Mentors have typically faced and overcome different challenges and can provide insights into how to turn obstacles into opportunities. Mentors can also help keep momentum and motivation going, especially during challenging times. Lastly, being a mentor is important for personal growth and reflection. Mentoring can help me reflect on aspects I have not considered, and this can lead to valuable insights and growth.

Laura: I believe the majority of my successes thus far have been down to having great mentors who have facilitated my ability to work in what can be a stressful fast-paced environment. Having supportive, unbiased mentors can be the difference between giving up and thriving forward. With dyslexia and a physical impairment, I am only too aware of the adversity faced in daily life and importance of peer support. I have learnt how important it is to create a supportive working environment such that the maximum potential of each individual can be achieved, thus engendering ‘in-lab’ discoveries which can translate to real life applications. As a result mentorship, and additionally advocacy, are hugely important to me because I felt like these aspects were lacking throughout my school to undergraduate to PhD journey. I am sure many others have felt like they are working things out by themselves. It seems down to luck and being in the right place at the right time that I have made it this far; I believe mentoring can assist with more intentional decision making. That’s why promoting STEM to the next generation with a focus on encouraging outreach and accessibility, especially for women and those underrepresented in STEM, and the promotion of chemistry to the next generation is hugely important to me.

Magda: While a typical journey of a graduate student used to be associated with a significant amount of isolation, stress and unrealistic supervisor expectations, we are now in a good position to stimulate changes that will positively influence the retention, success and the impact of researchers in the STEM field. This change does not need to come solely from the supervisors, in fact, I do advocate for catalyzing this process by graduate students. Therefore, we need to recognize the importance of cultivating the mentorship as the main pillar of academic activities and be ready to dedicate time to seeking and interacting with the mentors, and being mentors to others. I want to strongly encourage the graduate students to explore the opportunities to become mentors themselves. Becoming a mentor helps to be an informed, agile and future-ready mentee,

well positioned to understand mentors' standpoints, propose other techniques or approaches for mentorship and align expectations. I also encourage to proactively seek for various mentors, outside of our research teams or institutional structures. An excellent example of such support is the ACS Mentorship Program, which allows to connect 1-to-1 to experienced ACS members, willing to share their expertise in specific job markets (academia/industry), processes (such as transitioning into an independent research position or successful retirement), and most importantly their passion in seeing the long-term growth and success of their mentees. I believe that the choice of mentor's network can be best guided by appreciating of our own goals and boldest ambitions. Just believe in yourself!

Maha: As a beneficiary of mentoring, I strongly believe that mentors and role models can reduce barriers for underrepresented minorities (URMs) in STEM fields. Below is an example of my path to Stanford graduate school from being an international exchange student at the University of Mississippi (UM).

During my senior year of undergraduate studies in Pakistan, my hard work led to a U.S. Department of State exchange scholarship at UM, where I faced a new set of challenges as an international student and a young Muslim woman. I felt ashamed of my cultural and religious identity and was one of the few women in my engineering classes. During this tough period, I met Prof. Chen, a minority professor in ChemE, who championed me at UM. With his mentorship, I received the highest score in his thermodynamics class and initiated a research collaboration between UM and my Pakistani university, which became my Senior Honors Thesis. My UM experience exemplifies the power of diversity and mentorship in academia and the need to create an academic culture where all students feel comfortable. As a future professor, I want to strive for this goal and emulate Prof. Chen's example in effectively mentoring marginalized students.

Silvina: I am a people's person. As much as I enjoy my passion for physical sciences, I also enjoy—and feed off—social interactions. But, for me, social interaction has to serve a purpose. During my two plus decades of being a student, I learned a very simple equation: social interaction + education = mentorship. The purpose (education) was stimulated by social interaction (my mentors). This symbiotic relationship became key to appreciate and understand its importance when it comes to mentorship. Simply put, mentorship served a purpose for my growth as a professional with the aid of more experienced professionals.

At the conclusion of my dissertation defense mid-pandemic back in March of 2021, my principal investigator (PI) very eloquently concluded his congratulatory remarks with the following quote: "It takes a village". There is absolutely no way for a scientist to have a successful career if he/she was not mentored. Whether it is an unwelcome and mediocre or enriching and conductive mentorship experience, it served a purpose. Like any relationship, I find mentorship essential to my professional development. In a scientific setting, it is a perfect blend of social interaction and transferred technical knowledge.

Stanna: I would not be on the career path that I am today without phenomenal mentors that brought the best out of me and actively encouraged and helped me pursue my career goals. As

someone who comes from a rural, low-socioeconomic background and nearly failed out of organic chemistry during my undergraduate studies, I am fortunate to have had excellent mentors.

Scientific study necessitates that we remain open to new hypotheses and ways of thinking; indeed, we propose and actively explore hypotheses from a variety of angles in an attempt to obtain as full a picture as possible for a given system. It seems ironic, then, that many in the scientific community are not more invested in efforts to increase diversity, equity, and inclusion to promote a variety of perspectives on science as a whole (although this argument is often made to incentivize interest in DEI efforts, it should be noted that *everyone* should be supported and feel as though they have a place in science, regardless of whether the 'bottom line', or scientific output, is affected). While improvements have been made and are ongoing, there is a dire need for effective, inclusive mentorship in academia. Too many trainees are forced to leave their programs or feel trapped because mentors/advisers have abused the inherent power dynamic of academic settings. Mentorship is important to me because there were many times on my scientific journey that I was ready to give up and leave, but I had mentors that helped me keep going. At times, I have wondered where I would be without them. I wonder about those that do not have supportive mentors, and the institutions that tout impressive research but foster poor mentorship at the expense of trainees.

Julio: Navigating grad school and academia is not a simple task, especially for first-generation scientists/academics like many of us. We are also often not living in our home countries and not working in our mother tongues during grad school, so we end up living in a completely new world in which we have no experience. The role of a mentor to me is to provide guidance and assistance to the mentee, helping them climb the steps and in a certain way leveling them with their peers. This is where mentorship is the most significant to me when mentors help mentees navigate the difficulties of the academic world so that they can be their full selves and dedicate themselves to doing the best in their careers.

Salma: Postgraduate students typically come from different educational and socio-cultural backgrounds. As such, their journey through graduate school and integration into research life never takes a linear trajectory, but rather a combination of arcs, loops, and terrains. This makes it impossible to cover different learning needs through structured curricula alone. Mentorship is an excellent tool that can succeed where other tools fail. For instance, a mentorship relationship holds the potential to facilitate a meaningful exchange of ideas on life and career-related subjects that may prove challenging to broach within other types of academic relationships. In such cases, a mentor acts as a guiding force who provides valuable insights into navigating complex professional landscapes and offers constructive feedback and advice to help mentees overcome challenges and achieve their goals. The depth of these interactions and nuances of experiences shared often inspires growth and development for both parties. Furthermore, the benefits of mentorship extend beyond the academic realm; it also fosters growth in personal and professional spheres by opening doors to new networks, providing opportunities for career advancement, and enhancing mentees' self-confidence and esteem. The same outcomes might not be easily achieved through a typical supervisor-student relationship, where the inherent power dynamic and plausible conflict of interest might be a cause of distance, hesitance, or resistance, which could adversely impact such exchanges.

2. What have been some impactful mentorship strategies we have used or experienced?

Progna: As an early career researcher, I have found that one of the most impactful mentorship strategies is active listening. When I am mentoring a student or junior colleague, I make sure to listen carefully to their concerns, ideas, and goals. This allows me to provide guidance and support that is tailored to their individual needs, rather than simply giving generic advice.

Another important strategy is setting clear expectations and goals. I make sure that my mentees understand what is expected of them, and what they can expect from me as their mentor. This includes setting clear timelines, goals, and objectives, as well as regularly checking in to see how they are progressing.

In terms of my own experience as a mentee, I have been fortunate to have mentors who were supportive, encouraging, and willing to invest time and effort in my professional development. They provided me with opportunities to grow, challenged me to think creatively, and offered constructive feedback that helped me improve.

I believe that effective mentorship involves building strong relationships based on trust, respect, and mutual understanding. By listening carefully, setting clear goals, and providing guidance and support, mentors can help their mentees to reach their full potential and achieve their goals."

Elisabeth: Just like every relationship a mentorship is something highly individual. It needs to match the personalities, needs and abilities of the personalities involved. Nevertheless, I think there are some basic requirements such as trust, approachability and responsibility that will be part of every effective mentorship, and there are strategies which help to fulfill these:

- Having planned and spontaneous exchange between mentee and mentor. Planned meetings are important to structure the mentorship and to make sure you keep in contact, but often spontaneous meetings are much more helpful to solve problems when they are pressing or to talk about issues in a less formal atmosphere.
- Talk about your mentorship. Make clear what you expect from each other and what your respective responsibilities in your mentorship are. Find ways to communicate when you want something to change in your relationship.
- Talk about the mentee's plans for the future, how to get there and break it down into concrete tasks. Mentors are particularly important in transitioning phases of the mentee's career. The mentor can help to identify concrete and realistic goals as well as potential threats and shortcomings. It helps to write these points down and re-evaluate in your next meeting.
- Take inspiration from the corporate world. As scientists we usually stress that we do things differently than in the corporate world and that our ecosystem needs more freedom and less structure. While we often consider the more formalised leadership in companies as too hierarchical, some of the there established tools can be very helpful. For examples (bi)annual evaluation meetings can be useful to monitor progress and identify future focus points of the mentorship and it can be very helpful to use the SMART model when formulating goals.

Ernest

a. Strong, respectful and unbossed working and mentoring relationship
b. Active Listening and open to diverse perspectives
c. Collaboration and knowledge sharing
d. Nurturing environment & encouraging/fostering Diversity, Equity and Inclusion
e. Offering constructive feedback and challenging opportunities for growth & development beyond comfort zone

Helena: I, fortunately, had the opportunity to work in different research groups, which were very good working environments overall. Of course, no place is perfect, as there is always a place for improvement. As a young professional who wants to lead my own group in the future, I believe that a good strategy is to critically evaluate the working environment where you are and how people interact to try to absorb the positive points and try not to reproduce the negative ones.

While mentoring, it is important to be aware that every person is unique and that mentorship must be adaptable to their needs. Some students, for instance, may require more guidance and attention, while others are more independent. Some may have the best family support and structure to achieve their goals, while others may face unthinkable adversities that might impact their performance. A good mentor should be aware of these nuances to give guidance in a more personalized way.

Gabriele: In my opinion, one of the most effective mentorship strategies is leading by example. Throughout my career, I have always admired my mentors in many aspects, which pushed me to believe in them and in my own abilities. Their acceptance of failure, inspiring resilience, and willingness to listen to my problems and concerns have been precious lessons and memories for me. I feel grateful for the chance to learn by simply observing my mentors' attitudes. If I am who I am today, it is mainly because I followed in the footsteps of my mentors, who showed me the way.

As a mentor myself, I not only try to transfer what I have learned during my journey so far, but I also do my best to allow my mentees to express themselves: their personality, character, and attitude. From this perspective, a brainstorming session is a perfect environment where everything can be shared in a safe space. A brainstorming meeting always provides an opportunity to get to know people better, and understand their train of thought, and their mindset, which creates a human connection beyond a simple scientific plan. As a mentor, I always try to form a bond with those around me through empathy, as I have realized that it is more important to remember that we are human beings, regardless of any other label we may give ourselves.

Kevin: In my experience, some of the most impactful mentoring has come from informal discussions and observations rather than from formal mentoring events or meetings. For example, my PhD advisor challenged me to think about my career and the bigger picture of how science works in ways that were initially very unnatural to me. However, in hindsight, it was incredibly valuable. Additionally, reflecting on how different researchers do science or lead their groups has been helpful, especially when discussing these observations with mentors.

When it comes to making career decisions, I've found that having someone to bounce ideas off of can be extremely beneficial, even if the decision ultimately falls on me.

I've also found that mentoring others has helped me grow in many ways. Trying to transfer knowledge and help others has allowed me to reflect on my own experiences and gain a better understanding of where the mentee is coming from, including their goals and past experiences.

In my opinion, an important part of mentoring is understanding the mentee's perspective and goals. This helps tailor advice to the specific needs of the mentee and can lead to more effective mentorship. I did not find a silver bullet strategy that I can always use – I find mentoring to be highly individual and that I need to adjust my approach with every mentee.

Laura: A good mentor to me is someone who can listen and direct you to a solution or to the next step without pushing any particular agenda. They can be anyone but career-wise it has been helpful to me to have mentors one (or a few) steps ahead.

I am a mentee within a group established by the Women in Supramolecular Chemistry support group who connected myself and (at the time) 3 other PhD students with a mentor, we are all in the same field so there is a common ground but at different institutions such that we can discuss topics openly without judgement. This highly effective mentoring group began in 2019 and we have been meeting every month for 3 years (and continuing). Although the group has evolved, I still regard this group as my most effect professional support group. I think the key to our successful ongoing mentor-mentee relationship results from having clear expectations and boundaries, including how long and how frequent the meetings will be; we commit to 1 hour per month and make sure we never run over time, not only is this a manageable length of time to commit to but also ensures we stick to topic and have a productive meeting.

I believe it is also necessary to manage your own expectations of your mentors; know that they will not (and should not) solve all your problems for you, and that everyone has formed their opinions based upon their own experiences and biases etc. My most effective mentors (and what I try and do as a mentor to other) know when to listen and when to give advice appropriately. Small group mentoring can be particularly good because more diverse perspectives are bought to the discussion.

Finally, being proactive with any 'homework' that results from mentoring can lead to a much more productive mentor-mentee relationship in which no one feels like it was a waste of time, and then follow up with results (e.g. doing the job application, having a conversation etc.).

Maha: Impactful mentorship strategies I have experienced below:

1) Consistent one-on-one meetings: I have consistent one-on-one meetings with my graduate advisors, and these are one of the most effective things in my graduate school. My advisors have always given me the liberty to set my own research agenda. These meetings are extremely sacred for me as I use them to set small research milestones for myself. I also sometimes use them as brainstorming sessions with my advisors. There were a handful of meetings when we only discussed the problems, I was facing in data-analysis and how to best address those issues. These check-ins have also taught me how to prioritize and manage multiple research projects.

2) Effective communication: Any strong relationship starts with effective communication. Communication that is open, respectful, healthy, honest, and frequent. As a mentee, I have learned a lot from the communication styles of my advisors. Effective communication also helps resolve conflicts and creates a positive working environment for everyone involved.

3) Compassion, kindness, trust, and admiration: As a mentee, I can say with 100% conviction that my mentors have deep compassion and kindness towards me, and this has inculcated trust in me for them. I trust their research advice. They trust my research work. When there is trust, you listen to each other. I think these qualities are extremely important in an effective mentorship relationship.

   The last quality I will list is admiration. By admiration, I mean looking up to your advisor's work/research with regard. I look up to my advisors as my role models in science and this has shaped my personality as to how I envision running my own research group one day.

4) Effective feedback: Effective feedback is: direct, clear, and timely. For example, when my advisors provide me direct and clear feedback on my manuscripts and presentations, I know what they are looking for and how best to implement their feedback. Additionally, they set realistic timelines for providing feedback which are mostly two weeks for first review. This direct, clear, timely, and frequent feedback has been instrumental in my development as a first author on research projects.

5) End-of-year assessment: I think it is important to set milestones and then look back at these after a set period. My end-of-year assessments with my advisors have been very helpful in reflecting on my achievements, and areas of improvements the prior year and then using those to prioritize goals for the next year. "

Sai:

1) Setting clear expectations: An effective mentoring relationship begins with a strong foundation. A key element of building that foundation is having a clear on-boarding procedure for mentees. Similar to how a company would welcome a new employee, mentors should create a welcoming and professional environment for their mentees. This is especially important in academic settings, where new students and postdocs can feel overwhelmed and unsure of how to navigate their new roles and responsibilities.

   The on-boarding process should begin with an orientation session where the mentor provides an overview of the lab or research group's culture, values, and expectations. This session should cover the mentor's expectations for the mentee's work, including the expected timelines, communication protocols, and feedback mechanisms. The mentor should also provide information about the resources and support available to the mentee, such as lab equipment, software, and funding opportunities.

   Another important aspect of the on-boarding process is setting clear goals and objectives for the mentoring relationship. Mentors should work with their mentees to develop a shared understanding of what the mentee hopes to achieve during their time in the lab or research

group. This could include specific research projects, skill-building opportunities, or professional development goals.

2) Encourage independence: Effective mentoring involves more than simply providing guidance and advice to students and postdocs. It is a complex process that requires the mentor to be attuned to the needs and goals of their mentees, and to have a clear understanding of how to provide the support and guidance they need to achieve their objectives. One of the most important aspects of effective mentoring is the need to encourage responsibility in students and postdocs. By empowering them to take ownership of their work and to manage their time effectively, mentors can help to build trust and foster a sense of independence that is essential for success.

   Encouraging independence means giving students and postdocs the freedom to find their own pace and to work in a way that suits their individual learning styles and goals. This means not micromanaging their work or constantly checking in on them to see what they have accomplished. Instead, it involves setting clear expectations and goals, providing regular feedback and support, and trusting them to take responsibility for their own progress. This can be challenging for some mentors, especially those who are used to more hands-on approaches, but it is essential for building trust and fostering independence.

   One way to encourage responsibility is to involve students and postdocs in the development of research projects and goals. This means setting clear objectives, but also giving them the freedom to explore their own ideas and to pursue research questions that interest them. This can help to motivate them and to give them a sense of ownership over their work, which can be a powerful motivator for success. Additionally, providing regular feedback and support, such as offering guidance on research methodologies, reviewing drafts of papers, and providing constructive criticism, can help to build confidence and encourage independent thinking.

3) Finding the right fit: Lab work can be challenging and demanding, requiring dedication, long hours, and attention to detail. Mentors need to understand that not everyone is cut out for lab work, and it's not a failure to acknowledge that. Therefore, it's crucial to find a good fit between mentors and their students or postdocs.

   Mentors need to understand the strengths, weaknesses, and personalities of their students and postdocs. This information can be obtained through discussions, observation, and regular feedback. When mentors know their students' personalities, they can identify the right approach and communication style that works for each student. Some students may prefer more hand-holding and constant check-ins, while others prefer autonomy and independence.

   Mentors should encourage students to rotate in different labs to find a good fit. This approach can help students identify the kind of research they are interested in and the type of work environment they thrive in. Mentors should also allow their students to pursue their interests and passions. When students are working on projects that they are passionate about, they are likely to be more productive and engaged.

Finding a good fit between mentors and their students or postdocs is beneficial for everyone involved. When the fit is right, students are more likely to be engaged and productive, and mentors are more likely to enjoy working with their students. It also helps to build trust and respect between mentors and their students or postdocs.

4) Embracing diverse perspectives: In recent years, there has been a growing recognition of the importance of diversity and inclusion in academic and professional environments. American universities have made significant efforts to increase the enrollment of students from underrepresented groups and underprivileged backgrounds. The result is that the students in the labs are starting to reflect that trend. This shift in the student body's demographic makeup requires a corresponding shift in mentorship practices to ensure that everyone feels accepted and welcome.

   A good mentor should be aware of the challenges that students from diverse backgrounds may face and should take proactive steps to address them. Mentors should strive to create an inclusive environment that values diversity and promotes equity. This means creating an environment where students from different backgrounds can thrive and where their unique perspectives and experiences are valued. By doing so, the mentor can ensure that all students have equal access to opportunities and resources.

   Additionally, it is essential for mentors to be aware of the financial challenges that many students face. Graduate students, in particular, often need a fellowship to afford their programs. A good mentor should do everything they can to help their mentee secure funding. This could mean connecting them with funding opportunities, providing them with advice on how to write a grant proposal, or introducing them to potential funders. By doing so, the mentor can help ensure that their mentee can focus on their research and academic pursuits without the added stress of financial concerns.

   Another way that mentors can embrace diversity is by encouraging their mentees to pursue interdisciplinary research. This involves bringing together individuals from different fields of study to collaborate on research projects. Such collaborations can bring together a range of perspectives, allowing for a more comprehensive understanding of complex issues. Moreover, interdisciplinary research has been shown to lead to more innovative solutions to problems. By encouraging their mentees to pursue interdisciplinary research, mentors can help promote diversity in academic research and foster a more inclusive and collaborative environment.

5) Forging a personal connection: Mentors should make a point to meet with their mentees on an individual basis regularly. These meetings provide an opportunity for mentors to build a rapport with their mentees, discuss their academic progress, and offer guidance and support. Moreover, mentors should also be open to discussing personal issues with their mentees. While the primary focus of these meetings may be on academic and career-related matters, personal conversations can be valuable for the mentee's overall well-being.

   It is common for university students to experience depression, anxiety, and other mental health issues. The pressures of academic work, combined with the challenges of navigating

the transition to adulthood, can be overwhelming for some students. As such, it is crucial for mentors to be aware of the mental health challenges that students may face and be available to provide support.

In these one-on-one meetings, mentors should make it clear that they are available to listen to their mentees' personal concerns. By creating a safe and supportive environment, mentors can encourage students to open up about their problems. Once the students feel comfortable enough to share their personal issues, mentors can provide guidance and advice or refer them to appropriate resources such as counseling services.

Additionally, mentors can use these conversations to offer advice on work-life balance. It is essential for students to prioritize their mental health and well-being alongside their academic and professional pursuits. Mentors can offer suggestions on how students can manage their workload, develop good study habits, and practice self-care.

6) Embracing failure: As humans, we are all prone to making mistakes, and it is essential for mentors to acknowledge and share their failures with their mentees. Many times, students feel that they are the only ones struggling and that everyone else is achieving success effortlessly. By sharing their own failures and setbacks, mentors can create a supportive environment that encourages students to persevere despite setbacks.

   Sharing failures can also help students understand that failure is a natural part of the learning process. When mentors share their failures, they can explain how they learned from their mistakes and how they overcame the challenges they faced. This can help students develop a growth mindset and see failure as an opportunity for learning and growth rather than a setback.

   Furthermore, sharing failures can help mentors build trust with their mentees. When mentors share their own failures, they demonstrate vulnerability and authenticity. This can make students feel more comfortable opening up about their own struggles and concerns.

   It is important to note that sharing failures should be done in a constructive way. Mentors should not dwell on their mistakes or use them as an opportunity to vent. Instead, they should focus on the lessons they learned and how they overcame the challenges they faced. Additionally, mentors should be mindful of their mentees' emotional well-being and not share failures that may cause undue stress or anxiety.

Silvina: I have been on both sides of the spectrum--I have been a mentor (I taught 6th and 7th grade middle school science) and a mentee (during my graduate career). Some impactful mentorship strategies that I experienced as a mentee have been:

- Provided resources.
- Weekly 1 on 1 meetings.
- Feedback on both technical and soft skills.
- Room for creativity and discussion.

The aforementioned strategies are fundamental yet rather commonplace. One strategy that I know is not commonplace is liberty. I feel fortunate that my mentor was open to change the dynamic of the mentor-mentee relationship as I became a more "experienced" graduate student. As I became more independent, I started to set expectations for myself. It was a sense of freedom and creativity. Then, I would share my own expectation results with my mentor during our weekly 1:1 meetings. During my last four years of my graduate career, I decided to create a list of "Accomplishments in 20XX" and "Continue improving in 20X+1". For four consecutive Decembers, my mentor wrote a list of things "well done" that year and another list of things to "improve" the following year. This end-of-the-year assessment was instrumental to my growth as a young professional.

As a mentor, I have implemented the strategies my mentor used (above) for me. In addition to the ones I implemented on my own:

- Active listening.
- Set expectations.
- Guidance on professional and personal life.
- Constant communication, all plausible flatform.

To have a successful mentor-mentee relationship, it is important to be dedicated to your mentee ('active listening'). One needs to be truthful to oneself and genuinely provide positive feedback and constructive criticism, both in the professional and personal realm ('guidance on professional and personal life'). Thus, I believe it is important to be introspective and spend some time focusing on what one desires to achieve with the goal of having a mentee. Again, one needs to serve a purpose in a healthy, symbiotic relationship ('set expectations'). Frequency is key, so both the mentor and mentee have opportunities to follow-up and refresh expectations and materials discussed ('constant communication').

Lastly, to achieve effective mentorship, we need to have a long-term purpose, with the goal to grow endlessly. Quoting Barrette-Ng et. al, work, "commitment to mentorship is a commitment to lifelong learning within an academic micro-community." [Barrette-Ng, N., Nowell, L., Anderson, S. J., Arcellana-Panlilio, M., Brown, B., Chalhoub, S., ... & Wilcox, G. (2019). The mentorship guide for teaching and learning. Taylor Institute for Teaching and Learning Guide Series.]"

Olivia: Something I learned early on in my PhD program is that sharing moments of vulnerability as a mentor can have long-lasting effects on the outlooks of your mentees. When I began learning new data analysis methods as a first-year graduate student, I felt overwhelmed. As I watched Brandon—a senior PhD candidate who became my mentor—effortlessly scan lines of code and explain what they did, I couldn't help but think that I would never, could never reach that point of expertise. If brains are sponges, mine was oversaturated with new knowledge and unable to retain anything new. One day, while Brandon and I stared at my computer screen, my carefully guarded anxiety that I wasn't cut out for this type of research snuck out. I don't remember exactly what led up to this moment, but I clearly remember that Brandon looked at me knowingly and said, "You'll get there. First year is like drinking out of a firehose." He was months away from defending his PhD, but five years earlier, he had felt just like I did? That seemingly simple statement was empowering. I wasn't falling behind; I was right where I needed to be in my PhD journey.

Over the next several years, I had somehow accrued enough knowledge to become the go-to person for early-stage graduate students who had questions about the data analysis software. Whenever I

sat down with a student to look at their data, I made an effort to tell them what I had been told, that learning to use the software was like drinking from a firehose. I remember one student's facial expression immediately relaxed when I said that, much like I'm sure mine did three years before.

After finishing my PhD, I switched from studying chemistry via telescope observations to working in a lab for the first time in more than five years. I felt much like I did during my first year of grad school. I wasn't even going to apply for the fellowship to work in a lab because I clearly was not qualified to do experiments, but some nudging from my PhD advisor convinced me to give it a try anyway. In the first week or so of my appointment, my postdoctoral advisor (who had made a similar switch of fields) told me how she understood such a big change could be overwhelming and that I could reach out to her to talk through it if I needed. That type of vulnerability was again empowering, especially coming from someone in a supervisor role. In the past, scared to show that I knew less than everything, I had kept lists of questions about concepts I didn't understand to try to find answers on my own time. But knowing the expectation was that I started out knowing very little about this new project, I felt confident enough to ask questions in real time, and my learning has been much faster because of it.

In research, we place so much emphasis on people being "experts" in something that I think we sometimes forget that we can't be experts in all the things. Reminding mentees that they aren't expected to know everything (or perhaps even anything) when they start out can help them feel more secure in their pursuit of knowledge, empowering them to spend their energy on stumbling through research rather than on pretending they are going through the process flawlessly. Instead of perceiving asking questions as a sign of weakness, mentees can see asking questions as an opportunity for growth.

Stanna: Some of the best mentors I have had are excellent listeners. Whether I was describing a time I struggled, or an action that I believe they could have done differently, I have had mentors not only listen, but value what I was sharing.

The "hidden curriculum" of academia can be hard to navigate without a mentor. Throughout my journey, I have had mentors share resources and knowledge with me; as I advance, I compile and share information as well. I continually make myself aware of opportunities and resources, knowing that although I may not have all the answers as a mentor, I can hopefully point a trainee in a direction that will help them.

Julio: Impactful mentorship for me happens when the mentor-mentee connection is somehow personal, meaning that they acknowledge each other not only as professionals but also as individuals. The mentee needs to feel comfortable expressing their background and personality, and this is when they are their true selves and exceed in what they do. Of course, this is easier when the mentor is comfortable and open about themselves. As an LGBTQ+ scientist, I was lucky to find mentors who were allies, with whom I could always be myself and express my identity and personality. We know that LGBTQ+ scientists are less likely to be retained in STEM than their non-LGBTQ+ colleagues (DOI 10.1126/sciadv.aao6373) and that LGBTQ+ scientists are more productive and successful when they can express their sexual and gender identity in the work environment (DOI 10.1042/BIO20200024). I am certain that the support and confidence that I have always had from my mentors played and still play an essential role in my retention and

achievements in STEM and academia. As a mentor, I aspire to build a relationship of trust and respect with my mentees and acknowledge their personality, diversity, and uniqueness, as this is from my experience the foundation for a truly impactful mentorship experience.

Salma: Effective mentorship relationships require trust, mutual respect, and clear communication between mentors and mentees. They also require certain established protocols, a clear definition of boundaries and an understanding of roles, expectations, responsibilities and goals. These elements contribute to the foundation of successful mentorship relationships that are focused on development and growth, while also creating a safe space for mentorship.

I have been fortunate enough to engage in a diverse range of mentorship styles at different stages of my personal and professional career, with each relationship bringing forth unique experiences and perspectives that responded to different needs and played a vital role in shaping who I am today. Having a female mentor allowed me to be more vulnerable and realistic as I plan my career aspirations. Male mentors on the other hand helped me build my self-confidence while focusing on being more productive and professional. Alternatively, in-person interactions facilitated the development of long-lasting and robust relationships with my mentors and junior mentees, whereas virtual mentoring provided opportunities to connect with accomplished individuals possessing innovative viewpoints that may have otherwise been challenging to access.

My experiences taught me to appreciate the mentee-initiated mentorship, where I took initiative in seeking guidance and support from seasoned professionals, as well as the mentor-initiated relationships in which seniors and more experienced mentors took me under their wings to nurture my skills and help me advance in my career. I have mostly appreciated structural mentorship, which was facilitated through formal programs and involved pre-defined roles, programmatic goals, and set timelines. I found that the mentee-initiated style often suits more extroverted individuals who are confident in initiating conversations and building relationships. Analogously, effective mentor-initiated relationships require mentees to be more receptive and open-minded, with a willingness to learn from their mentors and appreciate their guidance, commitment and time. Comparably, the structural style can suit either personality. It might however be more restricted by time and requires mentors and mentees to regularly review and assess the progress made in their relationship and make adjustments where necessary.

3. How can we create a chemistry community that values and prioritizes effective mentorship?

Progna: Creating a chemistry community that values and prioritizes effective mentorship is crucial for the success of the field and the growth of the next generation of scientists. One way to achieve this is by recognizing and celebrating exceptional mentorship. Institutions can incentivize and reward mentors who demonstrate effective mentoring by providing grants, fellowships, or awards. Moreover, it is important to provide mentorship training to both new and experienced faculty members to ensure they are well-equipped to be effective mentors.

Another key strategy to create a culture of effective mentorship is by promoting an environment that values collaboration and communication. Chemistry departments can organize seminars, workshops, and events that encourage meaningful interactions between faculty, students, and

postdocs, and offer opportunities for mentor-mentee relationships to develop. Establishing a network of mentors and mentees within the department can also be helpful in providing a supportive community and encouraging mentorship from various perspectives.
Lastly, it is important to provide students and postdocs with the resources and support they need to excel. Creating a supportive environment can be achieved by offering workshops on professional development, financial planning, and work-life balance, as well as providing access to counseling services and support groups. By prioritizing effective mentorship and creating a supportive community, we can ensure that the chemistry field continues to grow and thrive.

Elisabeth: Being a good mentor is a skill that needs to be developed. As many aspects of leadership, until now it is undervalued in our education and curricula. That not only makes it less likely for people to recognize that effort needs to be invested in order become a good mentor, but also makes it less realistic that they find time and energy to actually do that. With mentoring programs becoming more and more common, it might be time to also implement programs in which potential mentors get time, support and resources to develop their skills.

In the scientific community we still have the ideal of the genius scientist who can do it all on their own and "just like that" develops the perfect solution to whatever problem they face. Sometimes highly successful scientists talk about how much they enjoy being a mentor, but only very rarely it occurs that they talk about their experiences as mentees, about the times when they struggled and needed some support to progress. We all can contribute to a change of culture by doing that ourselves, talking openly about the problems we had and how we could benefit from the experience and support of others. This will give evidence of the importance of mentorship and help younger scientists to understand how many puzzle pieces and contributions are needed for a good scientist to develop. Equally important it will reduce stress and imposter syndrome by lowering the imaginary bar to a more realistic height.

Ernest: I believe creating a chemistry community that values and prioritizes effective mentorship starts with awareness on the importance and benefits of effective mentorship to both parties involved. As I've previously mentioned, I believe mentorship is a two-street, so I think educating and informing the chemistry community about the importance of mentorship not only to the mentee but also to the mentor is a good way to begin creating a community that values its impact. Another thing to consider is providing mentorship training courses and resources to all parties involved to prepare and educate everyone about the goals and expectations of effective mentorship for a rewarding and positive experience. Academic conferences, symposia and networking opportunities should have a component on effective mentorship, collaboration and engagement as part of their program. Lastly, fostering a nurturing, diverse, and unbossed environment where everyone is valued and feels comfortable to share their perspective is crucial in creating a sustainable community for effective mentorship.

Helena: The first thing to do is discuss mentorship itself. Mentorship strategies are not typical subjects that are brought up and debated or trained on how to do it best. Fortunately, these points are being increasingly discussed, and mentorship training is being offered and encouraged in several universities. Even more important than providing training to the mentors is allowing mentees to share their feedback safely, without fear of retaliation. Mentor-mentee must be,

indeed, a two-way relationship, and both parties should be open to this exchange for it to succeed.

People usually associate mentors with people very close to us or our work/project, but sometimes having input from someone outside this circle might be beneficial to give and get unbiased advice. Therefore, beyond someone's personal network, virtual platforms have been developed and encouraged by some societies and communities to facilitate such exchanges between different mentors and mentees. Getting involved in these new ways of mentoring can be enlightening for both parties.

Gabriele: I believe we can create a chemistry community that prioritizes effective mentorship by promoting constant communication between mentees and mentors. In particular, evaluating mentees' performance is essential to help them improve their skills and attitudes. Advertising courses on this topic would be a valuable way to create connections and improve working relationships. In fact, giving mentors and mentees the right tools to communicate effectively by sharing opinions and providing feedback would dramatically increase productivity and research quality in both academic and industrial settings. Other opportunities, such as mental health counseling or psychological support, without stigmatizing or downplaying individuals' discomfort, would open up a new way of seeing researchers and workers, propelling a cultural revolution, and making effective mentorship a natural consequence of this shift.

Moreover, I believe it is important to inform and train people to use social media professionally. Gaining expertise on how to approach social media would not only be important for scientific communication but also to acknowledge and express different opinions in the STEM community.

To conclude, we can create a more accountable chemistry community centered on mentorship by building an empathic connection across different generations of scientists, which will lead to a more united and reliable scientific society.

Kevin: In my experience, formal programs that assign mentors to Ph.D. students can be a good starting point, but it is not a complete solution to effective mentorship.

One impactful strategy is to emphasize how being a mentor can contribute to personal growth. Mentoring provides an opportunity to reflect on one's own experiences and can lead to new insights and perspectives.

Another strategy is to start mentoring early, even in undergraduate programs. For example, having students mentor younger students can not only benefit the mentee, but also help the mentor develop valuable skills.

Finally, it may be beneficial to reward mentorship and make it part of the tenure evaluation process. This can help incentivize effective mentorship and encourage more senior researchers to invest in mentoring the next generation."

Laura: I believe systemic change in opinion will have to happen and then passed down to the next generation of leaders and mentors in order create a chemistry community that values and prioritizes effective mentorship. Despite some PIs having a bad reputation when it comes to

mentoring, students and postdocs may still want to work for them to benefit from the prestige that may come with a particular group. Not all believe that effective mentorship is critical for the success of research and therefore don't prioritise it. Unfortunately, this may result some people having a bad PhD experience, or even dropping out, and seeking mentorship outside of your institution may not be possible.

One strategy may be to prioritise collaboration over competition, at every level of academia; from members within the same group to research groups at different institutions. I believe this has to start with each individual research group fostering open communication and collaboration. Sharing knowledge, teamwork and group problem solving should be encouraged within a forum that allows everybody the opportunity to have a voice. This fosters a professional working environment in which members feel supported. I believe when the benefits of effective mentorship acknowledged, such as increased productivity, the leaders within the academic chemistry community will be encouraged to carry this attitude forward.

Maha: There are very few URMs in my field of research and a way to address this issue is their effective mentorship. I think it is extremely important to draw awareness to this issue in academic circles. An idea is to have a session or two on this topic at the American Chemical Society Fall Meetings. It will be great to see chemistry researchers around the world listen to each other's mentoring experiences and develop strategies for their mentor-mentee relationships.

Silvina: A chemistry community that prioritizes mentorship must show utmost importance towards mentor-mentee relationships. Although counterintuitive, "making" science should be placed in the "backburner", prioritizing those first- and second-year graduate students.
In addition,a well-developed and strategized system must be already in place, implemented by curricula. Also, I believe that an effective mentorship is one that prioritizes communication; thus, frequency in meetings is primordial. One-on-one meetings are key to discuss both technical and professional development progress. "

Olivia: When I was working on my PhD, students at my institution frequently requested that the department provide mentorship training to faculty. The idea was not welcome, at least at the time, for several reasons: it was expensive, it was unnecessary, it was even insulting (because faculty wouldn't be hired if they weren't qualified to be mentors). Instead, how faculty learned about mentorship seemed to be limited to pulling from one's personal experiences of what worked and what didn't work, which is strongly dependent on the person. (There were, however, a few people who sought out mentorship training on their own!) In response, students suggested that committee meetings include portions during which the mentor was required to leave the room to mirror portions during which mentees were required to leave during discussion. In these aspects absent of the mentor, students would be able to raise concerns about mentorship practices and get feedback on how to address said concerns. Again, the idea did not gain traction.

A chemistry community that values and prioritizes effective mentorship needs to have opportunities in place for mentors or future mentors to engage in mentorship training and receive feedback. Effective mentorship, like any other facet of research or teaching, is a skill that requires learning and practice. These are not easy things to implement, even with departmental support, because they come with added time requirements for faculty and (perceived and real)

risks for graduate students. Effective training requires dedicated time beyond a single seminar, for example. Graduate students and faculty peers who would provide feedback on mentorship practices, especially in small research groups or departments, may fear retaliation and decline to provide feedback on areas that would benefit from changes. To prioritize effective mentorship, we need to shift to a growth mindset in which developing an effective mentorship is seen to benefit everyone (e.g., by giving graduates better outcomes, increasing research productivity, having a more collaborative and collegial environment versus a disproportionately skewed power dynamic) rather than as a criticism of how people lead.

Stanna:

1) Creating/providing access to formal training opportunities: It is intriguing that although much of a PI role involves management (of trainees, research projects, funding, etc.), this skillset is often not a significant criteria for the hiring process. Furthermore, formal avenues for obtaining management training in a scientific setting are not as widely known or accessible. Much of a PI's management training is done "on the job," and herein lies the dissonance–the criteria valued at the time of hiring does not fully encompass what the role actually requires, and trainees pay the price for this inexperience. Institutions need to value mentorship when hiring, but also support mentors in obtaining training for these skills.

   Additionally, graduate students and postdocs are usually expected to train other graduate students or undergraduates without any guidance on how to do so; having formal training or best practices for this type of mentorship could mitigate harm done by ineffective mentoring.

2) Increased accountability: Academic institutions often claim that they prioritize effective mentorship, but all too often harbor and promote mentors/PIs that display recurring patterns of harm/abuse; the power imbalance is skewed such that trainees are often reluctant or afraid to give feedback, and even when they do, institutions are slow to act on it, if at all. If we want to create a community that values and prioritizes effective mentorship, we need to demonstrate that it is something to be taken seriously and acted upon, and not merely hollow statements without any accountability.

Julio: Although most of us become mentors at some point in our careers as chemists, we are not trained for it and are many times not aware of good practices. I believe the chemistry community needs to acknowledge mentoring as an essential skill, especially in academia (where we all spend many years of our lives and when we need mentors the most). We need to share good and bad experiences and highlight their impact on the mentees' careers. I believe quantitative research that draws relationships between mentorship experiences and mentees' career paths could be an effective way to highlight this impact and get the community to better value and prioritize effective mentorship.

Salma: By promoting and encouraging mentorship, STEM communities can foster an environment that supports the growth and success of all members. This can ultimately lead to increased diversity and inclusivity within the STEM community, as well as better-prepared professionals who are equipped with valuable skills and knowledge to contribute to the

advancement of scientific knowledge and technological innovation. Engaging in academic discussions regarding the advantages of fostering functional mentorship alliances, particularly for marginalized individuals within the STEM field, constitutes an initial measure. Senior STEM professionals should be encouraged to lead by example and serve as role models for mentorship in the community. Mentors should also be encouraged to communicate the advantages they acquire by mentoring others. Ultimately, this would help counteract the prevalent notion that only mentees benefit from mentorship relationships. Such action can also stimulate increased involvement of other mentors in similar activities.

Analogously, incorporating productive mentorship connections into STEM education as an essential component, from the high school level onward, could potentially foster significant progress within the community. This would also help students and EMCRs gain a clear understanding of the distinctions among supervisory, mentorship, and training relationships and their respective expectations.

Encouraging and facilitating mentorship programs, along with establishing accolades that incentivize mentoring endeavors among diverse professional groups and organizational departments, should be given significant attention as well. Moreover, creating opportunities or platforms to evaluate the effectiveness of mentorship programs and providing feedback to mentors could fortify the implementation of functional mentoring alliances.

4. Anything else you would like to share?

Progna: As an early career researcher, I have grown both personally and professionally while cultivating mentorship and diversity ideals and practices. My growth as a scholar and mentor has been closely tied to my values of inclusivity, diversity, and support, and I am constantly striving to create a lab community that reflects these ideals. Initially, I had a traditional approach to mentorship, where I would provide guidance and support to students and postdocs in the lab. However, I realized that my role as a mentor goes beyond technical guidance and extends to the personal and professional development of my trainees. I actively seek to create a supportive and inclusive lab environment where everyone feels valued, heard, and supported. This involves being available to my trainees, providing feedback on their work, and encouraging them to pursue their interests and goals.

Additionally, I have recognized the importance of diversity and inclusion in the lab and have implemented practices that prioritize equity, such as creating opportunities for underrepresented students and fostering a culture of respect and inclusivity. I have also worked on developing a mentorship network that extends beyond the lab, connecting trainees with mentors and resources that align with their interests and career goals.

Laura: Resources (That I am aware of/have used – sometimes you can’t find a mentor but other resources can be just as useful throughout a PhD)

Mentoring:

- The Royal Society of Chemistry: https://www.rsc.org/careers/cpd/mentoring/

- Women in Supramolecular Chemistry: Mentoring | Women in Supramolecular Chemistry (womeninsuprachem.com)
- The Thesis Whisperer: The Thesis Whisperer

Books:
- “You Are Not a Fraud: A Scientist's Guide to the Imposter Phenomenon” By: Dr Marc Reid. You Are Not a Fraud: A Scientist's Guide to the Imposter Phenomenon — Marc Reid, PhD (dr-marc-reid.com)
- “Managing your mental health during your PhD” By Dr Zoe Ayres. Home | Mental Health in Academia (zjayres.com)

Websites:
PhD planning & Viva prep:
- Thinkwell: Tools to give to your student (ithinkwell.com.au) & PhD Toolkit (ithinkwell.com.au) & PhDVivaQuestions.pdf
- Viva Survivors: Viva Survivors – Daily viva help for PhDs (viva-survivors.com)

Planning Reactions:
- Reaxys: https://www.reaxys.com/#/login
- CAS SciFinder: https://scifinder-n.cas.org
- Master Organic Chemistry: https://www.masterorganicchemistry.com
- LibreTexts Chemistry: https://chem.libretexts.org/Ancillary_Materials/Demos_Techniques_and_Experiments/General_Lab_Techniques
- Not Voodoo X.4: http://www.chem.rochester.edu/notvoodoo/
- The Schlenk Line Survival Guide: https://schlenklinesurvivalguide.com/
- Chemistry Views (Chemistry European Journal): Tips and Tricks for the Lab: Column Choices – ChemistryViews & Tips and Tricks for the Lab: Column Troubleshooting and Alternatives - ChemistryViews
- Data Organic Chemistry: NMR Spectroscopy :: 1H NMR Chemical Shifts (organicchemistrydata.org)
- Chemtips: Chemtips | Because Organic Chemistry is Hard Enough (wordpress.com)

Dealing with Literature:
- Feedly: https://feedly.com/i/my
- Genie: genei | AI-powered summarisation & research tool
- Endnote: EndNote
- Zotero: Zotero | Your personal research assistant
- Mendeley: Mendeley - Reference Management Software

Other:
- ACS Reviewer Lab: https://institute.acs.org/courses/acs-reviewer-lab.html
- ACS webinars: https://www.acs.org/content/acs/en/acs-webinars.html
- My Green Lab: https://www.mygreenlab.org/ambassador-program.html
- Scientific Writing: I focus and write | Scientific Writing Online Courses

Silvina: To my scientific community, whether you are an undergraduate student, starting your career or a tenure professor, close to retirement - is to become a mentor. Mentoring relationships are extremely fulfilling, where both the mentor and mentee learn from each other. My advice is to nature and appreciate this unique symbiotic relationship.

Author biographies

Jacky Deng (University of Ottawa)

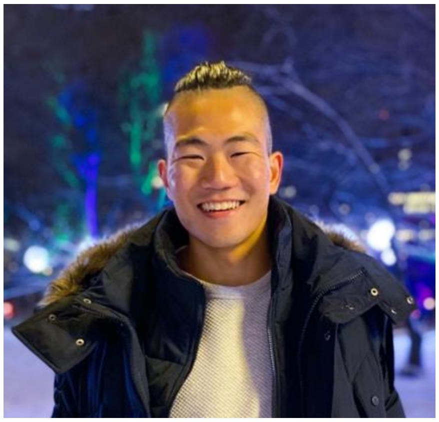

Jacky Deng (he/him) is a PhD Candidate working with Prof. Alison Flynn at the University of Ottawa (uOttawa). Jacky's research focuses on the experiences of learners who speak English-as-an-additional language in chemistry, with implications for how individuals and institutions can better support learners from diverse backgrounds in science. As a graduate student, Jacky has published peer-reviewed articles and book chapters in chemistry and science education (*e.g.*, *Journal of Chemical Education*, *International Journal of Science Education*), and has presented at various local, national, and international conferences and seminars. Jacky was selected to be a 2022 CAS Future Leader and was the inaugural J.C. Tito Scaiano Graduate Student of the Year at uOttawa in 2021. He was also a recipient of a SSHRC Vanier Scholarship in 2021—Canada's most prestigious graduate scholarship. Outside of his research, Jacky has been engaged in various national initiatives and committees to improve equity, diversity, and inclusion (EDI) in Canadian chemistry and higher education, including work conducted with the Canadian Society of Chemistry's Working for Inclusivity, Diversity, and Equity (WIDE) Committee and the Society of Teaching and Learning in Higher Education's (STLHE) EDI Task Force. He loves teaching and was selected as the 2020 Teaching Assistant of the Year by uOttawa's Science Students' Association.

Progna Banerjee (Argonne National Laboratory)

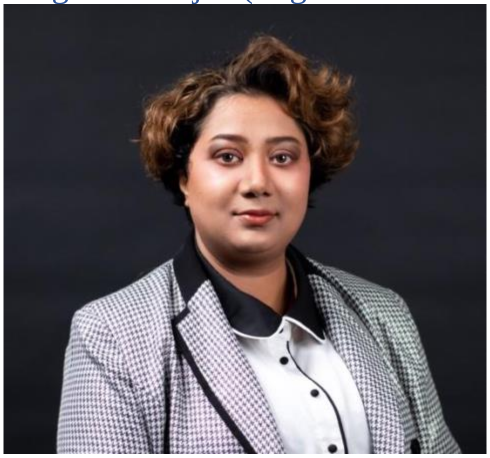

Progna Banerjee is a postdoctoral appointee at Argonne National Laboratory, where she works on interdisciplinary research in experimental condensed matter physics, materials chemistry, and electronics. She received her Ph.D. in Physics with Materials Chemistry and M.S. in Strongly Correlated Condensed Matter from the University of Illinois at Urbana-Champaign in 2018 and 2014, respectively, after earning her M.Tech. in Solid State Technology and M.Sc. in Physics from the Indian Institute of Technology, Kharagpur.

During her Ph.D., Progna used a template-based synthetic technique called "cation exchange" in chalcogenide nanocrystals from quantum dots to establish their unusual structural arrangement, which caused exhibition of room-temperature superionicity and topological insulating properties. At the University of Texas, Austin, where she was an NSF MRSEC postdoc fellow, she investigated light-matter interactions in bioinspired nanostructures. She has led materials sub-teams and worked on multi-university research projects with a focus on understanding the mechanism of light interaction in leafhopper-insect derived nanostructures called brochosomes with inherently high antireflective properties ascribed to their buckyball-like structures. While at the Lawrence Berkeley National Laboratory, where she was an EBI-Shell ALS affiliated postdoc fellow, she worked on battery science.

Currently, Progna is part of an interdisciplinary team at Argonne National Laboratory looking to discover nanomaterials using Artificial Intelligence/Machine Learning assisted colloidal synthesis and post-synthetic cation exchange combinatorics using an autonomous liquid-handling platform. Her research focuses on the expansion of the nanomaterials library into unconventional metastable phases and studying the emergence of various physicochemical properties in these systems.

Progna's interdisciplinary research background, particularly in the areas of experimental condensed matter physics, materials chemistry, and electronics, gives her a unique perspective in her field. She has authored several publications in Nature and ACS family journals, and has received recognition for her work, including awards for her research dissemination & academic leadership efforts, and presentations at international conferences.

Elisabeth Kreidt (Technical University Dortmund)

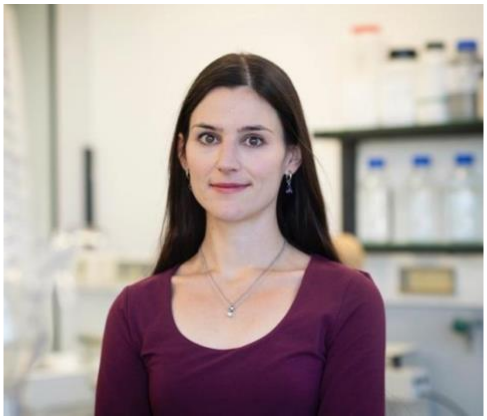

Already during her undergrad studies Elisabeth got fascinated by the unique physical properties of the lanthanoids and she then decided to study them in depth during her PhD. In the group of Michael Seitz she developed new functionalised lanthanoid cryptates, a class of structurally particularly well defined and stable lanthanoid complexes. Apart from using these special complexes for the realisation of a molecular nanocode she studied their photophysical and magnetic properties, including their circularly polarised luminescence. To broaden her knowledge in organic chemistry and to learn more about the toolbox about supramolecular chemistry she then joined the group of Dave Leigh in Manchester for her postdoc. There she realised and studied molecular knots of the size of small peptides and worked on the realisation of a chemically fueled unidirectional molecular motor. She then secured the scholarship of the state of North Rhine-Westphalia for scientists returning to Germany and since early 2022 she is setting up her independent research group. In her work she combines her knowledge about lanthanoid coordination chemistry, the physical properties of lanthanoids and supramolecular chemistry to develop systems which allow to dynamically control and manipulate the photophysical and magnetic properties of the lanthanoids. Aim of this work, among others, is to develop new tools for (bio)medical research, but also to improve the fundamental understanding of these properties. Elisabeth was elected a 2022 CAS future leader and is member of the young college of the North-Rhine-Westphalian academy of sciences and arts.

Ernest Awoonor-Williams (Novartis Institutes for Biomedical Research)

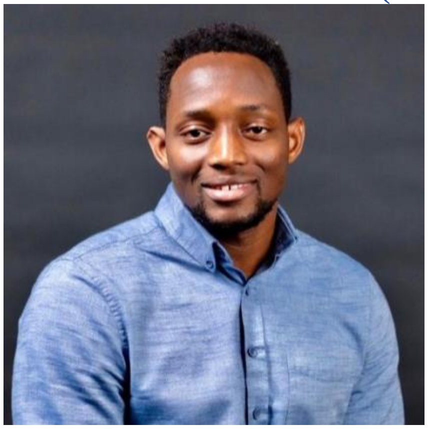

Ernest completed his B.Sc (Hons) degree in Chemical Physics at Mount Allison University under the supervision of Prof. Khashayar Ghandi in 2014. Ernest's honors research focussed on muonium free radical chemistry in green solvents, particularly supercritical carbon dioxide. This research combined both computational and experimental approaches, with the latter carried out with researchers at the TRIUMF National Laboratory in Vancouver, BC. In September 2014, Ernest moved to St. John's, NL to pursue graduate studies in Chemistry at Memorial University of Newfoundland. He completed his Ph.D. in Computational Biochemistry in May 2020 under the supervision of Prof. Christopher Rowley. His PhD research explored the use of computational methods in modeling the covalent modification of druggable cysteines in enzyme targets. As a Ph.D. student, Ernest was a visiting research scholar at the University of Ottawa studying enzyme inhibition by targeted covalent inhibitors under the supervision of Prof. Jeffrey Keillor. During his Ph.D. Ernest was awarded the prestigious NSERC Vanier Canada Graduate Scholarship, IUPAC Young Chemist award and an Advanced Research Computing Fellowship. In 2021, Ernest joined the Novartis Institutes for BioMedical Research (NIBR) in Cambridge, MA, as an Innovation Postdoctoral Fellow. At NIBR, he is exploring in silico approaches towards drug discovery, such as the impact of mutations in drug binding and pathogenesis.

Helena Mannochio Russo (University of California San Diego)

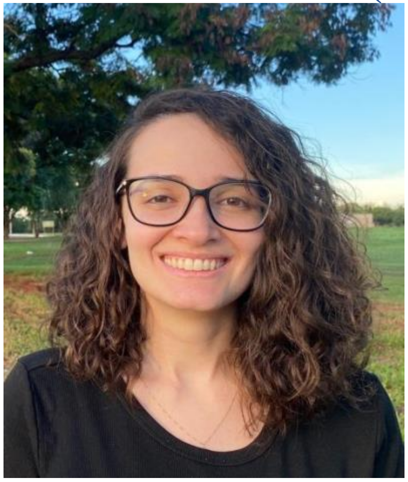

Helena Mannochio Russo received her B.Sc. degree in Chemistry from the University of Campinas (2015) and her M.Sc. and Ph.D. in Chemistry from São Paulo State University (2018,

2022). She carried out two internships at the University of Geneva, the first during her undergraduate studies and the second during her master's. During her Ph.D., she was a visiting scholar at Skaggs School of Pharmacy and Pharmaceutical Sciences (University of California San Diego), funded by the Fulbright Commission. During her undergraduate and master studies, she specialized in the phytochemistry of Brazilian plants. During her Ph.D., she performed mass spectrometry-based metabolomics and chemosystematics studies of Brazilian plants and investigated natural products with larvicidal activity against vectors of tropical diseases. Currently, she is a postdoctoral researcher at the University of California San Diego in Prof. Dorrestein's group, where she is interested in mass spectrometry repository-scale analyses, data science, and the chemistry of natural products. Helena was selected to attend the 71st Lindau Nobel Laureate Meeting (dedicated to Chemistry, 2022) as well as elected a 2022 CAS Future Leaders by the American Chemical Society.

Gabriele Laudadio (Scripss Research Institute)

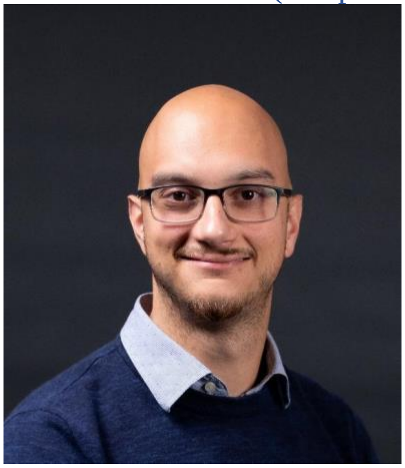

Gabriele Laudadio was born on July 12th 1991 near Teramo (Italy). He received his master degree in Organic Chemistry from the University of Pisa in 2016, under the supervision of Prof. Adriano Carpita. From May 2016, he started a Ph.D. project in the Micro Flow Chemistry and Synthetic Methodology at the Eindhoven University of Technology (Netherlands), under the supervision of Prof. Timothy Noël. The primary focus of his research was the application of Green Chemistry to improve Organic Chemistry methodologies, exploring the possibility to obtain molecules of interest faster and in a more efficient way, combining Continuous-Flow microreactor technology with electrochemistry and photochemistry. After a brief period as postdoctoral fellow and group leader in Prof. Noël's group at the Van't Hoff Institute for Molecular Sciences in the University of Amsterdam, he joined Prof. Baran's group at The Scripps Research Institute as a Hewitt Foundation Fellow. At Scripps, he is exploring the electrochemical functionalization of heteroarenes, to generate C(sp3)-C(sp2) bonds in a faster and efficient way. His contribution on flow chemistry and electrochemistry was recognized with several awards, including the CAS Future Leaders (2022), the IUPAC-SOLVAY International Award For Young Chemists (2021) and the IUPAC-Zhejiang NHU International Award for Advancements in Green Chemistry (2021).

Júlio Terra (Swiss Federal Institute of Technology)

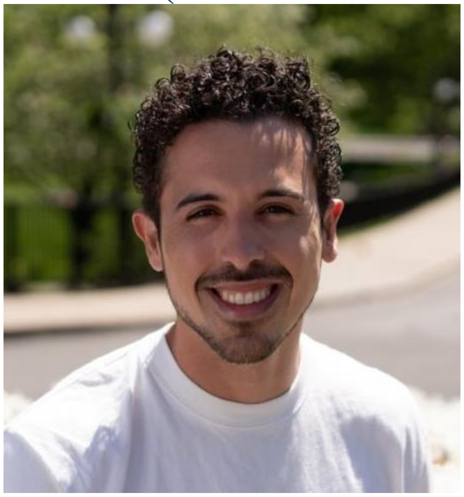

Júlio was born and raised in a village in the Brazilian countryside, he obtained his B.Sc. degree in Chemistry in 2015 from the Federal University of Lavras (Brazil) with an undergraduate exchange period at the University of Toronto (Canada) in 2012-13, when he worked at the laboratory of Professor Bob Morris. In 2016, Júlio obtained his M.Sc. degree from the Federal University of Minas Gerais (Brazil) under the supervision of Professor Flávia Moura. He then joined the group of Professor Audrey Moores at McGill University (Canada) to pursue his Ph.D. degree in Green Chemistry and Catalysis, which was obtained in 2023. During his Ph.D., Júlio worked on the synthesis and nanodesign of silica-based structures that were used to make catalytic processes greener and more sustainable by exploring photocatalytic properties and nanoconfinement effects. Júlio is now a Banting post-doctoral fellow at the Swiss Federal Institute of Technology in Lausanne (Switzerland), where he designs nanocatalysts using atomic layer deposition to be used in biomass valorization processes. Júlio is also passionate about science education and equity, diversity, and inclusion in STEM. In addition to chemistry, Júlio is also passionate about Brazilian music and loves playing the guitar and singing; he also enjoys dancing, exercising, hiking, traveling, and learning new languages.

Kevin Jablonka (Swiss Federal Institute of Technology)

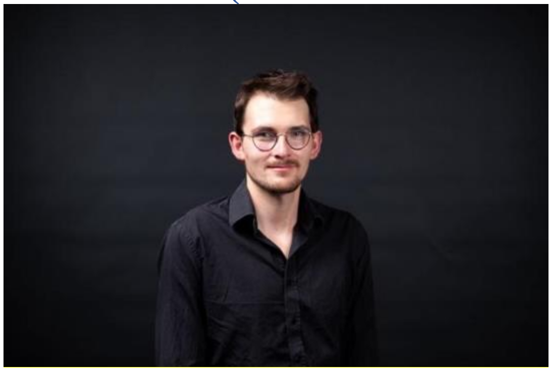

Kevin Jablonka was born in 1996 in a small town in Germany and spent a lot of time in the lab while in high school. He scored successes in student science fairs such as “Jugend forscht”, which he is now supporting as juror, or the International Conference of young scientists.
After high school, he obtained his bachelor’s degree in chemistry at TU Munich and then joined EPFL for his master’s studies in chemistry and an extended study degree in applied machine

learning. While at EPFL, he also spent half a year in the Quantum Chemistry and Modeling department of BASF in Tarrytown, New York.

Kevin continued pursuing computational research during his Ph.D. in the group of Berend Smit, in which he focused on accelerating materials discovery across scales. got involved in the cheminfo ELN ecosystem. He also developed a toolbox for digital (reticular) chemistry and is co-leading the ChemNLP open science project, which aims at creating a foundational chemistry model. He now leads a research group at the Helmholtz Institute for Polymers in Energy Applications (HIPOLE).

Laura Bickerton (University of Edinburgh)

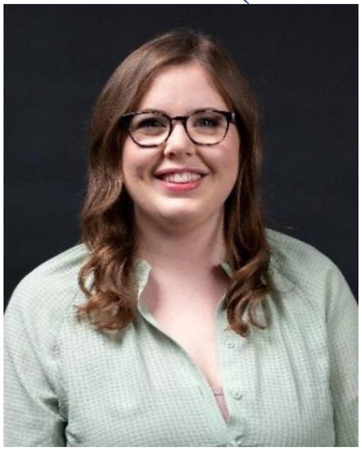

Laura Bickerton is currently undertaking a 3-year EPSRC funded postdoctoral position working within the Prof Paul Lusby group in collaboration with Prof Guy Lloyd-Jones (University of Edinburgh) and Prof Fernanda Duarte (University of Oxford) on metal-organic cages for catalytic applications. Laura completed her BSc (Hons) in Biochemistry and Biological Chemistry at the University of Nottingham in 2018, and subsequently completed her DPhil in Inorganic Chemistry at the University of Oxford under the supervision of Professor Matthew Langton and Professor Paul Beer. During her PhD research she designed, synthesised, and tested supramolecular anion receptors, capable of facilitating the transport of biologically relevant anions across cell membranes, including the incorporation of halogen bonding and stimuli-responsive anion transporters. Laura's PhD research resulted in 4 first author publications, 3 poster prizes at the RSC Macrocyclic and Supramolecular Chemistry Meeting (2019, 2021 and 2022), and subsequently being named as a CAS Future Leader in 2022. Additionally, Laura is a mentee within the WISC (Women in Supramolecular Chemistry) mentoring network and is a mentor in association with the Royal Society of Chemistry. Now at the University of Edinburgh, she is a member of the postdoctoral committee working to ensuring a supportive working environment for all postdocs.

Maha Yusuf (Stanford University)

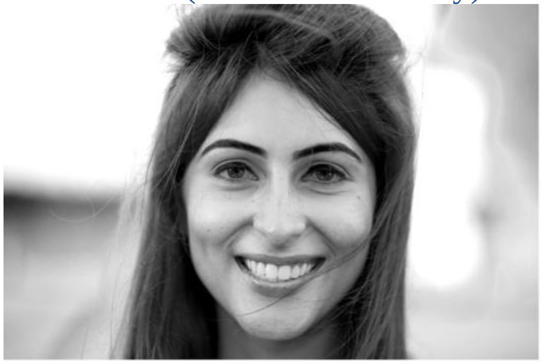

Maha Yusuf is an incoming Presidential Postdoctoral Fellow at Princeton University. She is currently finishing up her PhD in chemical engineering at Stanford University. In her thesis research, Maha has used high-resolution neutron and X-ray imaging techniques to investigate Li plating characteristics on graphite electrodes during extreme fast charging of lithium-ion batteries. During her PhD, Maha has led extensive beamtime experiments at national and international neutron and X-ray facilities: National Institute of Standards and Technology Centre for Neutron Research (NIST), Oak Ridge National Laboratory (ORNL), and Paul Scherrer Institut (PSI).

For her work, Maha has won awards including the 2022 American Chemical Society CAS Future Leader; 2020 Stanford Distinguished Student Energy Lecturer; and 2020 and 2021 ECS and American Institute of Chemical Engineers Travel Grants. Maha holds an M.S. in chemical engineering from Stanford (2017) and a B.E. from the National University of Sciences and Technology, Pakistan (2013). Prior to Stanford, Maha worked on oil and gas rigs as a drilling engineer for Schlumberger in amazon rainforests in Colombia. To date, Maha has published six peer-reviewed journal articles and holds two patents.

Saigopalakrishna “Sai” Yerneni (Carnegie Mellon University)

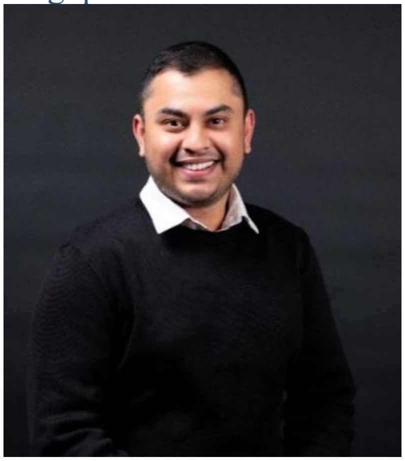

Saigopalakrishna “Sai” Yerneni earned a Bachelors in Technology from Anna University (India) in 2012 followed by a Masters’ in Biomedical Engineering from Carnegie Mellon University (CMU), Pittsburgh in 2015. He then continued at CMU as a Dowd Fellow to pursue his PhD in Biomedical Engineering. His doctoral work focused on understanding extracellular vesicle

mediated cell-to-cell communication in health and disease. Specifically, he developed biofabrication technologies to study extracellular vesicle biology in regenerative medicine, cancer and infectious diseases. Currently he is a postdoctoral fellow at CMU and is working on advanced drug delivery technologies for nucleic acid drugs and gene therapies.

Silvina “Silvi” A. Di Pietro (Lawrence Livermore National Laboratory)

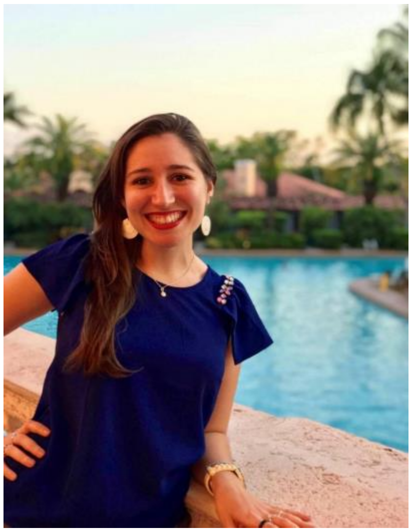

Silvina A. Di Pietro graduated from Florida International University (FIU) in the Spring of 2021 with a PhD in environmental chemistry. In June of 2021, she joined the Class of 2021 National Nuclear Security Administration (NNSA) Graduate Fellowship Program sponsored by Pacific Northwest National Laboratory (PNNL) as a post-doctoral scientist at Lawrence Livermore National Laboratory (LLNL). Upon successfully completing her 1-year post-doctoral appointment, she transitioned to a research position at LLNL. Currently, she is working under the supervision of Dr. Kiel Holliday within the Materials Science Division where she is working on developing additive manufacturing into a viable technology for nuclear applications.

During her graduate studies, she was a Department of Energy (DOE) Fellow at the Applied Research Center (ARC) and chemistry student within the Department of Chemistry and Biochemistry in FIU. At ARC, she assisted in development of ammonia gas injection for uranium remediation at the DOE Hanford Site in Washington State. Her dissertation project was titled *Uranium Fate and Mineral Transformations upon Remediation with Ammonia Gas*. The ammonia injection remediation technique has the potential to treat and sequester uranium, the major contaminant of concern released in the vadose zone at the Hanford Site. Silvina has been a recipient of the highly competitive 2022 CAS Future Leaders program sponsored by the American Chemical Society, the 2018 *Innovators in Nuclear Technology R&D* awarded by the DOE Office Nuclear Energy and 2016 *Roy G Post Scholarship* Foundation awarded by the Waste Management Symposia. Silvina’s professional interests include water chemistry, geochemistry, climate change, and remediation of contaminated areas. Her hobbies are traveling, dancing, playing tennis, visiting art museums, and painting.

Stanna Dorn (California Institute of Technology)

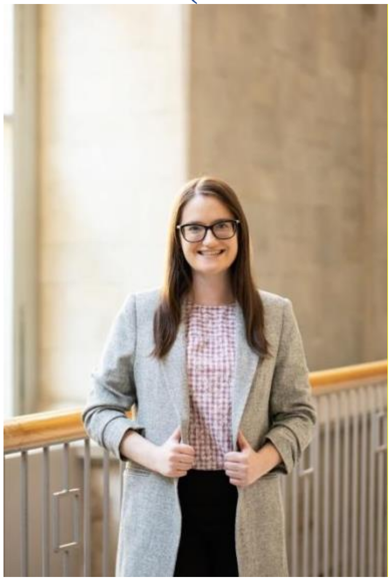

Stanna is an aspiring academic from Grayling, MI. She obtained a B.S. in chemistry and B.A. in music from Hope College, where she performed organometallic research with Prof. Jeffrey Johnson. She then received her PhD from Indiana University-Bloomington, where she worked with Prof. Kevin Brown on cooperative catalysis methods for alkene difunctionalization. Currently, Stanna is an NIH postdoctoral fellow in the Reisman lab at Caltech, working on total synthesis. Outside the lab, she enjoys reading, playing the flute, and proving that getting C's in organic as an undergraduate does not define one's future success in the field.

Olivia Harper Wilkins (NASA Goddard Space Flight Center)

Dr. Olivia Harper Wilkins, PhD, is a NASA Postdoctoral Program (NPP) Fellow in the Astrochemistry Laboratory at NASA Goddard Space Flight Center. In her current research, she primarily conducts cosmic ice experiments to understand how UV radiation affects interstellar, cometary, and planetary ices using the Sublimation of Laboratory Ices Millimeter/submillimeter Experiment (SubLIME). She also uses radio telescope observations to study the chemistry in high-mass star-forming regions. Dr. Wilkins earned her PhD in Chemistry at the California Institute of Technology (Caltech), where she conducted observational astrochemistry research as a National Science Foundation (NSF) Graduate Research Fellow. Before that, she earned a B.S. in Chemistry and Mathematics from Dickinson College and was a Fulbright Research Fellow in Germany. Besides research, Dr. Wilkins is involved in the NASA Goddard Association of Postdoctoral Scholars (NGAPS+) and is a coordinator for the NASA CONNECTORS (CONNECting high school students TO ResearcherS) program. She is also a Member-at-Large for the Maryland Section of the American Chemical Society (ACS) and an Affiliate of the National ACS Younger Chemists Committee (YCC). Dr. Wilkins was named a 2022 CAS (a division of the American Chemical Society) Future Leader and an AGU Science Activation (SciAct) Affiliate. She is passionate about science communication and using art to share science, something that culminated in her writing and illustrating *Astrochemistry* (2021) for the ACS's In Focus series. Dr. Wilkins enjoys traveling with her husband and son and spending time at coffee shops.

Salma Ahmed (The University of Queensland)

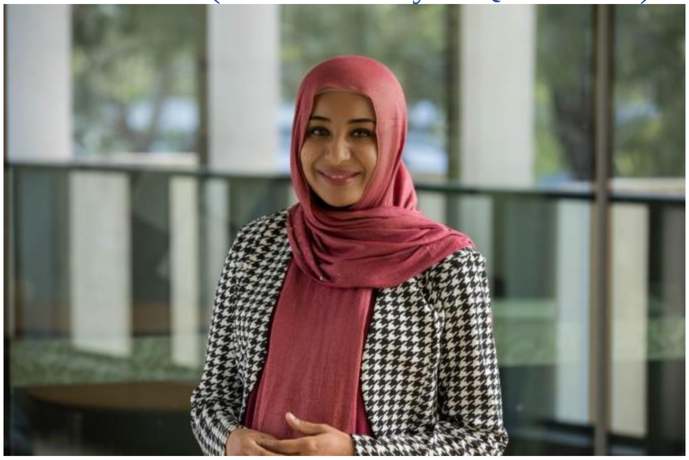

Salma Elgaili Ahmed is a final-stage PhD candidate of bioengineering under the supervision of Prof. Kristofer Thurecht at The University of Queensland, Australia. Her chemical engineering background combined with her MSc and PhD training inspired her to work in the interface of engineering and science to develop next generation tools for drug delivery. Salma has worked within highly interdisciplinary teams and developed myriads of nanocarriers for applications including precision cancer nanomedicine, diabetes treatment and miRNA delivery.

Salma is highly dedicated to her field. She has earned several accolades, including a CAS Future Leaders fellowship from the American Chemical Society, fully-funded MSc and PhD scholarships, and multiple other sci-com, travel and publication awards.

She is also a skilled communicator, where she has produced several high-impact publications and has presented her research at prestigious conferences and platforms. Salma is an active member of the wider community. She is an associate member of the Royal Society of Chemistry (AMRSC) and is dynamically involved in teaching and mentoring students in STEM fields. She was also a TEDxOmdurman Speaker, a TEDxUQ Curator and Mentor, and an accredited MHFAider.

As she approaches the end of her PhD, Salma is eager to embark on the next phase of her career. She is excited to apply her skills and knowledge to solve real-world problems in both academia and industry.
